# Low willingness to pay for pre-exposure prophylaxis (PrEP) among men who have sex with men (MSM) in China

**DOI:** 10.1186/s12889-020-08488-w

**Published:** 2020-03-16

**Authors:** Wangnan Cao, Shengzhi Sun, Liping Peng, Jing Gu, Chun Hao, Jibin Li, Dannuo Wei, Stuart Gilmour, Jinghua Li

**Affiliations:** 1 School of Public Health, Brown University, Providence, RI USA; 2 School of Public Health, Sun Yat-sen University, Guangzhou, China; 3 Sun Yat-sen Global Health Institute, Sun Yat-sen University, Guangzhou, China; 4 Department of Clinical Research, Sun Yat-sen University Cancer Center; State Key Laboratory of Oncology in South China; Collaborative Innovation Center for Cancer Medicine, Guangzhou, China; 5 Graduate School of Public Health, St. Luke’s International University, Tokyo, Japan

**Keywords:** Pre-exposure prophylaxis, Men who have sex with men, HIV prevention, Pay, Market rate, China

## Abstract

**Background:**

Pre-exposure prophylaxis (PrEP) is recommended as an HIV prevention strategy for key populations, in particular men who have sex with men (MSM). However, the willingness to pay market rate for PrEP is largely unknown. This study aimed to investigate the willingness to pay for PrEP and its associated factors among MSM living in Mainland China.

**Methods:**

A cross-sectional survey was conducted among 689 MSM who were recruited through a gay-friendly health consulting service center in Chengdu, China during 2018–2019. We collected information on participants’ willingness to pay for PrEP and its potential correlates (e.g., PrEP awareness and acceptability, perceived risk of HIV infection) using a structured questionnaire. Univariate and multivariate logistic regression were used for data analyses.

**Results:**

Only 14.1% of respondents indicated they would not pay any money for PrEP, around half (49.3%) would like to pay $14–84 per month, and very few (6.8%) would like to pay ≥283 per month (market rate). We found that PrEP awareness (unadjusted odds ratio (ORu) = 1.41; 95% CI: 1.01–1.97), acceptability (ORu =1.20; 95% CI: 1.07–1.34), perceived PrEP adherence (ORu =1.23; 95% CI: 1.08–1.41), and perceived PrEP benefit in reducing condom use (ORu =1.29; 95% CI: 1.07–1.55) were all associated with participants’ willingness to pay the market rate for PrEP. Other facilitators of PrEP pay willingness included full disclosure of sexual orientation to health professionals, high HIV literacy, and a high degree of HIV disclosure with sex partners.

**Conclusions:**

The overall willingness to pay for the market rate of PrEP was low among this urban sample of Chinese MSM. Programs aiming to promote PrEP pay willingness should provide enhanced counseling to improve PrEP-related cognition, deliver accurate HIV/PrEP information to increase health literacy, and decrease stigma towards sexual minorities to develop trust with health professionals.

## Background

Men who have sex with men (MSM) experience a disproportionate burden of HIV/AIDS disease in China [1]. The HIV prevalence among MSM ranged between 7 to 32% globally in a recent systematic review (2016) [2], but among Chinese MSM HIV prevalence has increased from 1% in 2005 to 7% in 2012 [1]. HIV prevalence is strikingly high in some Chinese cities, such as Chengdu (18% in 2013 [3]) and Kunming (17% in 2015 [4]).

Pre-exposure prophylaxis (PrEP) is regarded as a promising HIV prevention strategy, with a common treatment regimen consisting of a single pill composed of a compound of antiretroviral (ARV) drugs. There are three types of PrEP available, including daily oral PrEP, on-demand PrEP (refers to taking ARV before and after sex) and long-acting injectable PrEP (LAI-PrEP, refers to injecting long-acting drugs every 4 to 12 weeks). Given the demonstrated safety and efficacy of PrEP [5], the World Health Organization [6] recommends oral PrEP as an additional prevention strategy for key populations, and UNAIDS [7] has established a 2016–2021 strategy with an ambitious target of ensuring access to PrEP for three million people at substantial risk by 2020. In China, there is an ongoing multi-center trial among MSM (2018–2020, ChiCTR-IIN-17013762).

Along with these PrEP promotion programs, awareness of PrEP (11–58% [5, 8–12]) and willingness to PrEP uptake (64–92%) [13, 14] have increasing among MSM globally. The 2017 Chinese National Internet Survey among 4581 MSM showed that 22.4% of them had heard of PrEP and the majority (76%) would like to try PrEP [12]. In a recent study about half (46%) of 708 Chinese MSM were considered eligible for PrEP based on objective criteria (e.g., in a non-monogamous relationship, had condomless anal sex with a casual male partner in the past 3 months) [15], and 42% of 323 Chinese MSM self-perceived as appropriate PrEP candidates [16]. Literature reported that facilitators of PrEP uptake included risky sexual behaviors, high self-perceived HIVrisk, and possessing a high level of HIV literacy [17, 18], while barriers to PrEP uptake included worries about side effects, low positive expectations of its efficacy, and no experience of HIV testing [12].

Most of these acceptability studies and randomized controlled trials (RCTs) provided PrEP to the MSM participants for free, and participants’ willingness to pay for PrEP are rarely investigated. However, in the real world, PrEP is expensive and costs around $670 per month in overseas countries [19] and $212–990 per month in different regions of China [20, 21]. Costs associated with PrEP uptake include the prescription of ARV, and provision of PrEP counseling and adherence monitoring. The costs of tests (HIV, STIs, eGFR) would not be an issue as the current health care system in China provides free HIV/STIs testing for MSM, and the price for eGFR is cheap ($7–$14 each time). However, PrEP drugs are not covered so far by any of the existing health care and insurance systems in China, and the variation in cost of PrEP is affected by various factors, such as the local living expense (e.g., urban area is usually higher than rural area), source of the drug (e.g., hospitals usually charge higher than LGBT friendly non-governmental organizations). Poverty and economic constraints are identified barriers access to HIV prevention and treatment services, and the cost of TDF/FTC is a major barrier to PrEP implementation even in Western Europe’s high-income countries [22]. In settings where PrEP is considered unaffordable from a public policy perspective, studies of willingness to pay are important to understand the degree of private purchasing of PrEP.

Six quantitative papers and two qualitative papers have reported on the willingness to pay for PrEP among MSM [19, 23–27]. The majority of the MSM participants in these studies (65–77% in 2012–2015 [23–25]) were unwilling to pay or only willing to pay a small proportion of the full cost (e.g., 12% in 2014–2015 [19]). For example, an online survey among 1151 MSM reported that 77% of the participants were unwilling to pay the market rate of $340 for PrEP in 2014 [23] in Taiwan, China. PrEP-30, a fee-based program among 195 Thai MSM, showed that they were willing to pay for PrEP only when it was delivered at an affordable price (<$1 per day) [28]. Two qualitative studies (one among 19 young African-American urban MSM in 2009 and the other among 30 young MSM in Mississippi, US) reported that few of the participants were willing to pay for PrEP, and most participants felt that the cost would make PrEP inaccessible to them [26, 27]. Only one of these studies investigated factors associated with willingness to pay among MSM, and identified factors included the previous receipt of nonoccupational postexposure prophylaxis and positive attitudes toward PrEP [23]. To the best of our knowledge, no similar studies have been conducted among MSM living in Mainland China and factors associated with the willingness to pay for PrEP are poorly understood in the existing literature.

Therefore, the aim of the present study was to investigate the willingness to pay for PrEP and its factors among MSM living in Mainland, China. Two PrEP pay indicators were introduced as the outcome: pay or not (> 0 vs. 0), pay the market rate or not (≥ $283 vs. < $283 per month). In addition, we did a supplementary analysis (See Supplementary) using the generic drug market rate of ≥85 per month as the willingness to pay threshold [29]. Based on relevant published studies and the PrEP cascade, we hypothesized that trust in health professionals, risk perception of HIV infection, and PrEP-related cognition (awareness, acceptability, perceived PrEP adherence, and perceived PrEP benefit in reducing condom use) would be associated with the willingness to pay for PrEP among MSM.

## Methods

### Study design and setting

We conducted a cross-sectional survey among MSM who live in Chengdu, China between November 2018 and April 2019. We selected participants from this specific city as MSM residing in Chengdu are heavily affected by HIV [30]. There were no supporting policies and ongoing programs regarding PrEP uptake in Chengdu at the time of the study.

### Participants and recruitment

Participants were recruited from service users of a local gay-friendly health consulting service center, and we sampled potential participants from its contact list. This list includes clients who have agreed to be contacted for a research purpose, and only center staff have access to this list. To decrease the travelling burdens of the clients, most of the surveys were done when the clients visited the center for HIV testing. Meanwhile, center staff also made phone calls to clients in the contact list to invite them to come to the center to participate in this study. Two center staff were hired to facilitate this recruitment process.

Inclusion criteria for participants were: male older than age 18 years old and self-reported anal intercourse with at least one man in the last six months. A total of 890 eligible participants were approached, of whom 711 (response rate of 79.9%) completed the survey. Among these 711 MSM, we excluded 22 (3.1%) participants who self-reported themselves as HIV-positive, therefore, the final sample for the current analysis was 689. All these 689 participants had no previous PrEP uptake experience or were not on PrEP at the time of recruitment.

Participants were briefed about the study purpose in person at the center and then provided written informed consent before completing a self-administered survey questionnaire. The questionnaire took an average of 20 min to complete with an iPad, and completeness and logic errors were automatically checked. Participants were offered $7 cash to compensate for their time. Ethical approval was obtained from the ethics committee of Sun Yat-sen University ([2018] 049).

### Measures

The validity of measures used in the present study was ensured by a thorough literature review and pilot test. Before the formal survey, all measures were pilot tested in three separate samples, including 15 college students, 3 MSM peer leaders in the collaborating NGO, and 25 MSM who met the study’s inclusion criteria. Revisions were made based on the pilot results (e.g., delete overlapping items) and comments from these participants (e.g., shorten the item description, fix the awkward wording).

## Background

Socio-demographic information collected included age, ethnicity, education level, marital status, personal income, job, and local residence. We also asked the participants about sexual orientation and the age of first homosexual intercourse.

### Willingness to pay for PrEP

Willingness to pay for PrEP was collected by using the single item “If PrEP is not free, what is the maximum amount you are willing to pay for it per month (USD)?” Responses were 0, < 14, 14–28, 29–57, 58–84, 85–113, 114–141, 142–282, and ≥ 283.

### Health status and service utilization

#### Self-rated health and the history of sexually transmitted infection (STI)

We asked the participants to rate their general health status. Responses included very poor, poor, just fine, good, and very good. In addition, we asked the participants about their history of STIs.

#### HIV testing history and HIV status

We asked about the participants about their HIV testing history and HIV status (positive or negative or unknown) at the time of the survey. Each participant was offered a free HIV test at the collaborating organization after completing the survey to confirm their HIV status. The present analysis used their self-reported data on HIV status.

#### Disclosure of sexual orientation to health professionals

We constructed a 3-item scale to assess the participants’ disclosure status of sexual orientation to health professionals. Three questions were “Whether you have fully disclosed your sexual orientation to the hospital doctors?” “Whether you have fully disclosed your sexual orientation to the CDC officers?”, and “Whether you have fully disclosed your sexual orientation to the NGO peers?” Responses were yes (scored 1) or no (scored 0). An overall disclosure scale of sexual orientation to health professionals was calculated by summing up three responses, with a higher score indicating a higher degree of disclosure.

### HIV-related characteristics

#### Risk perception for HIV infection

For participants who were HIV-negative or serostatus unknown, participants were asked to rate their perceived risk of HIV infection in the next six months with a single item; responses were on a five-point Likert scale from 1 (very high) to 5 (very low).

#### HIV literacy

HIV literacy was assessed by a bespoke 12 item scale, which consisted of measurements on HIV prevention, transmission, recognition, and treatment. The scale has the advantage of including the most updated information on HIV, such as “*Undetectable (viral load) equals to untransmittable*” and “*Immediate ART initiation is recommended for newly diagnosed patients*”. Participants were asked to assess whether each item statement was correct or wrong. An HIV literacy score was calculated by counting the correct answers, with a higher score indicated a higher level of HIV literacy.

#### Sexual behaviors with partners

Participants were asked to recall the total number of sex partners in the past month and condom use behaviors with each partner. Participants who reported having sex with more than one partner in the past month were classified as engaging in multiple sex partnerships. Participants who did not use condoms for all sexual intercourse in the past month were classified as performing inconsistent condom use.

#### HIV status communication with partners

We constructed a 2-item scale to measure the participants’ HIV status communication with partners, “*I told my HIV status to all partners in the past month*” and “*I asked all partners about their HIV status in the past month*”. Responses were on a 5-point Likert scale (1 = never, 2 = occasionally, 3 = half of the time, 4 = most of the time, and 5 = all the time). An overall HIV status communication scale was calculated by summing up the two responses, with a higher score indicating a higher level of HIV status communication.

### PrEP-related cognitions

#### PrEP awareness

We self-constructed a 3-item scale to measure the participants’ PrEP awareness. Participants were asked whether they had heard of daily oral PrEP, on-demand PrEP, and LAI-PrEP. An overall PrEP awareness scale was calculated by summing up the three responses for these PrEP types, with a higher score indicating a higher level of PrEP awareness. After answering these three questions, we provided all participants a standard information sheet regarding the three types of PrEP, including names of the ARV involved, the method (oral or injection) and frequency (daily, on-demand, or every eight weeks) of usage, adherence and regular checkup requirements, potential benefits/risks, and the estimated cost. The interviewers went through this sheet with each participant.

#### PrEP acceptability

We self-constructed a 3-item scale to measure the participants’ PrEP acceptability. Participants were asked about their willingness to use the three types of PrEP respectively if it is free on a 5-point Likert scale (1 = definitely will not, 2 = probably will not, 3 = uncertain, 4 = probably will, 5 = definitely will). An overall PrEP acceptability scale was calculated by summing up three responses, with a higher score indicating a higher level of PrEP acceptability.

#### Perceived PrEP adherence

We self-constructed a 3-item scale to measure the participants’ perceived PrEP adherence. Responses to these questions were entirely hypothetical, given that all participants had no previous PrEP uptake experience and were not on PrEP at the time of recruitment. Participants were asked to rate their perceived adherence to the three types of PrEP respectively on a 5-point Likert scale (1 = very low, 2 = low, 3 = uncertain, 4 = high, 5 = very high). An overall perceived adherence scale was calculated by summing up three responses, with a higher score indicating a higher level of perceived adherence.

#### Perceived PrEP benefit in reducing condom use

Participants were asked “*Do you agree with the statement that you will reduce condom use (as a benefit) during anal sex after taking PrEP?”* Responses were on a 5-point Likert scale (1 = totally disagree to 5 = totally agree).

### Statistical analysis

Willingness to pay or not (> 0 vs. 0) and to pay the market rate (≥ $283 vs. < $283 per month) were analyzed as two dependent variables, respectively. Odds ratios for background variables and independent variables (e.g., HIV literacy, PrEP awareness) were first presented in the univariate analysis (ORu). The association between independent variables and the dependent variables were calculated adjusting for potential sociodemographic confounders, and presented as adjusted odds ratios (AOR) and their 95% confidence interval (95% CI). We finally fitted the multivariate models and presented adjusted odds ratios from the multivariate model as modelled ORs (ORm) with their 95% CIs. All statistical analyses were performed using IBM SPSS Statistics (version 25, SPSS Inc.), and two-tailed *p* < 0.05 was considered as statistically significant.

## Results

### Descriptive characteristics

Around half of the participants were aged 25 years or older and 57.3% attended college or above. Half (54.0%) of the participants were single, 13.4% were married (male-female marriage), and 26.0% were in a relationship with their boyfriends. The majority (75.9%) of the participants self-reported their sexual orientation as homosexual. Around half (54.9%) of the participants had their first homosexual intercourse before 21 years old.

The majority (81.7%) of the participants had tested their HIV status at least once before this survey, and 70.2% of the participants reported a high intention to test their HIV status in the next six months. Among the whole sample, 80.4% reported themselves as HIV-negative, and 19.6% reported themselves with unknown serostatus. Less than 10 % (7.8%) of the participants reported a history of STIs. Few (6.4%) participants had fully disclosed their sexual orientation to hospital doctors, 19.7% to NGO peers, and 41.9% to CDC officers. (Table 1).

**Table 1:** Background characteristics of the participants (*N* = 689)

Items	N % Mean ± SD	
Backgrounds		
Age (years old)		
18–21	118	17.1
22–25	193	28
26–29	123	17.9
30–33	91	13.2
> 33	164	23.8
Highest education obtained		
Middle school or lower	55	8.0
High school	102	14.8
Three-year college	137	19.9
Bachelor’s degree or above	395	57.3
Marital status		
Single	372	54.0
Married (male-female marriage)	92	13.4
Having boyfriends	179	26.0
Divorced/widow/others	46	6.7
Type of job		
Full time	438	63.6
Part time	30	4.4
Unemployed	221	32.1
Personal monthly income (USD)		
< 141	76	11.0
141–424	145	21.0
425–849	254	36.9
850–1132	100	14.5
1133–1415	51	7.4
> 1415	63	9.1
Self-identified sexual orientation		
Homosexual	523	75.9
Heterosexual	3	0.4
Bisexual	131	19.0
Others	32	4.6
Age of first homosexual intercourse (years old)		
< 17	68	9.9
17–18	123	17.9
19–20	187	27.1
21–25	189	27.4
26–30	52	7.5
> 30	70	10.2
Health status and service utilization		
Self-rated health status		
Poor/very poor	17	2.5
Just fine	198	28.7
Good/very good	474	68.8
The history of STI infection (Yes)	54	7.8
HIV status		
Negative	554	80.4
Unknown	135	19.6
HIV testing ever (Yes)	563	81.7
Intention to test HIV status in the next six months		
Low intention	205	29.8
High intention	484	70.2
Full disclosure of sexual orientation to hospital doctors (Yes)	44	6.4
Full disclosure of sexual orientation to CDC officers (Yes)	289	41.9
Full disclosure of sexual orientation to NGO peers (Yes)	136	19.7
*Overall disclosure of sexual orientation to health professionals*^*a*^*(0–3)*	0.68 ± 0.87	

*STI* sexually transmitted infection, *CDC* Centers for Disease Control and Prevention, *NGO* Non-governmental Organization.

^a^The Cronbach’s alpha coefficient was 0.534, and 1 factor explained 52.4% of the total variance;

The mean score for perceived HIV risk was 2.36 (SD = 1.09), ranging from 1 to 5. The mean score of the HIV literacy scale was 9.25 (SD = 1.98), ranging from 0 to 12. In the past month, the prevalence of multiple sex partnership was 30.8% and the prevalence of inconsistent condom use was 28.9%. Slightly more than 40 % (41.8%) of the participants told all partners their own HIV status all the time while 30.8% asked partners about their HIV status all the time.

Regarding the willingness to pay for PrEP, 14.1% would not pay any money for PrEP, 27.2% would like to pay ≥85 per month (generic drug market rate), and very few (6.8%) would like to pay ≥$283 per month (market rate). Regarding PrEP-related cognitions, the mean scores were 0.69 (SD = 0.80, range: 0–3) for PrEP awareness, 11.44 (SD = 3.41, range: 3–15) for PrEP acceptability, 12.27 (SD = 3.23, range: 3–15) for perceived adherence to PrEP, and 2.38 (SD = 1.55, range: 1–5) for perceived PrEP benefit in reducing condom use. (Table 2).

**Table 2:** PrEP and HIV related knowledge, cognition, and behavior (*N* = 689)

Items	N % Mean ± SD	
Willingness to pay for PrEP		
Amount willing to pay for PrEP per month (USD)		
0	97	14.1
< 14	66	9.6
14–28	139	20.2
29–57	118	17.1
58–84	82	11.9
85–113	33	4.8
114–141	50	7.3
142–282	57	8.3
≥ 283	47	6.8
Percentage (of personal income) of willing to pay for PrEP		
0	97	14.1
< 5%	193	28.0
6–10%	117	17.0
11–25%	154	22.4
> 25%	128	18.6
HIV-related characteristics		
Perceived risk of HIV infection (range: 1–5)	2.36 ± 1.09	
Perceived risk of STI infection (range: 1–5)	2.22 ± 1.10	
HIV literacy score ^a^ (range: 0–12)	9.25 ± 1.98	
Sexual behaviors in the past month		
Inconsistent condom use (Yes)	199	28.9
Engage in multiple sex partnership (Yes)	212	30.8
HIV status communication with partners		
*HIV disclosure scale to sexual partners*^*b*^ (range: 2–10)	6.95 ± 2.69	
PrEP-related cognition		
PrEP awareness		
Awareness of daily oral PrEP (Yes)	225	32.7
Awareness of on-demand PrEP (Yes)	205	29.8
Awareness of long injecting PrEP (Yes)	43	6.2
*PrEP awareness scale*^*c*^*(range: 0–3)*	0.69 ± 0.80	
PrEP acceptability		
Willingness to use daily oral PrEP (very likely/likely)	421	61.1
Willingness to use on-demand PrEP (very likely/likely)	540	78.4
Willingness to use LAI-PrEP (very likely/likely)	439	63.7
*PrEP acceptability scale*^*d*^*(range: 3–15)*	11.44 ± 3.41	
Perceived PrEP adherence		
Perceived adherence to daily oral PrEP (very high/high)	487	70.7
Perceived adherence to on-demand PrEP (very high/high)	580	84.2
Perceived adherence to LAI-PrEP (very high/high)	490	71.1
*Perceived PrEP adherence scale*^*e*^*(range: 3–15)*	12.27 ± 3.23	
Perceived PrEP benefit in reducing condom use (range: 1–5)	2.38 ± 1.55	

*STI* sexually transmitted infection, *PrEP* Pre-exposure prophylaxis, *LAI-PrEP* Long-acting Injectable PrEP;

^a^The Cronbach’s alpha coefficient was 0.626, and 4 factors explained 53.1% of the total variance;

^b^The Cronbach’s alpha coefficient was 0.786, and 1 factor explained 82.4% of the total variance;

^c^The Cronbach’s alpha coefficient was 0.356, and 1 factor explained 45.2% of the total variance;

^d^The Cronbach’s alpha coefficient was 0.820, and 1 factor explained 73.9% of the total variance;

^e^The Cronbach’s alpha coefficient was 0.832, and 1 factor explained 75.2% of the total variance;

### Factors associated with pay for PrEP

#### Associations between background variables and pay for PrEP

Personal monthly income was positively associated with participants’ willingness to pay for PrEP (ORu = 1.24; 95% CI: 1.04–1.46). Participants were less likely to pay for PrEP if they were older (ORu = 0.50; 95% CI: 0.31–0.79), reference: <=25), married (ORu = 0.31; 95% CI: 0.17–0.54, reference: single), and had first homosexual intercourse at an older age (ORu = 0.53; 95% CI: 0.34–0.82), reference: <=21). Education, type of job, and sexual orientation were not significantly associated with this willingness. No background variables were associated with participants’ willingness to pay $283 per month for PrEP. (Table 3).

**Table 3:** Background characteristics associated with pay for PrEP

Items	Pay		Pay $283	
	Row%	ORu (95% CI)	Row%	ORu (95% CI)
Age (years old)				
< =25	90.4	1.00	7.7	1.00
> 25	82.3	0.50 (0.31, 0.79)**	6.1	0.78 (0.43, 1.40)
Highest education obtained				
Below than university	83.7	1.00	6.1	1.00
University or above	87.6	1.38 (0.9, 2.12)	7.3	1.22 (0.66, 2.23)
Marital status				
Single	89.8	1.00	7.8	1.00
Married	72.8	0.31 (0.17, 0.54)***	4.3	0.54 (0.18, 1.57)
Having boyfriends	87.7	0.81 (0.47, 1.42)	7.3	0.93 (0.47, 1.83)
Type of job				
Full time	87.0	1.00	7.5	1.00
Part time	90.0	1.35 (0.40, 4.58)	3.3	0.42 (0.06, 3.21)
Unemployed	83.3	0.74 (0.48, 1.17)	5.9	0.77 (0.40, 1.49)
Personal monthly income	–	1.24 (1.04, 1.46)*	–	1.16 (0.94, 1.42)
Self-identified sexual orientation				
Homosexual	86.4	1.00	6.3	1.00
Bisexual	83.2	0.78 (0.46, 1.31)	8.4	1.54 (0.45, 5.31)
Age of first homosexual intercourse (years old)				
< 21	89.4	1.00	7.9	1.00
> =21	81.7	0.53 (0.34, 0.82)**	5.5	0.67 (0.36, 1.24)

†*P* < 0.10, *P < 0.05, **P < 0.01, ***P < 0.001;

*ORu* univariate odds ratio;

#### Associations between health status, service utilization and pay for PrEP

The overall disclosure of sexual orientation to health professionals was significantly associated with participants’ willingness to pay for PrEP in both univariate analysis (ORu = 1.35; 95% CI: 1.02–1.78) and adjusted analysis (AOR = 1.32; 95% CI: 0.99–1.77, *p* < 0.1) after adjusting for significant background variables. Self-rated health status, history of STIs, and HIV testing behavior and intention were not significantly associated with this willingness. None of the above variables was associated with participants’ willingness to pay $283 per month for PrEP. (Table 4).

**Table 4:** Univariate analysis on factors associated with pay for PrEP

Items	Pay			Pay $283	
	Row%	ORu (95% CI)	AOR (95% CI)	Row%	ORu/AOR (95% CI)
Health status and service utilization					
Self-rated health status	–	1.08 (0.80, 1.46)	–	–	0.98 (0.65, 1.49)
The history of STI					
No	85.5	1.00	–	6.8	
Yes	90.7	1.66 (0.65, 4.28)		7.4	1.10 (0.38, 3.19)
HIV testing ever					
No	84.9	1.00	–	8.7	
Yes	86.1	1.10 (0.64, 1.90)		6.4	0.71 (0.35, 1.45)
Intention to test HIV status in the next six months					
Low intention	85.9	1.00	–	5.4	
High intention	86.0	1.01 (0.63, 1.61)		7.4	1.42 (0.71, 2.84)
Overall disclosure of sexual orientation to health professionals	–	1.35 (1.02, 1.78)*	**1.32 (0.99, 1.77)†**	–	1.06 (0.76, 1.48)
HIV-related characteristics					
Perception of risk for HIV infection	–	0.94 (0.77, 1.14)	–	–	1.12 (0.86, 1.47)
Perception of risk for STI infection	–	1.00 (0.82, 1.22)	–	–	1.22 (0.94, 1.57)
HIV literacy scale	–	1.20 (1.09, 1.32)***	1.16 (1.05, 1.28)**	–	0.98 (0.84, 1.13)
Sexual behaviors in the past month					
Inconsistent condom use					
No	85.5	1.00	–	6.7	
Yes	86.9	1.13 (0.70, 1.83)		7.0	1.05 (0.55, 2.00)
Engage in multiple sex partnership					
No	86.4	1.00	–	5.9	
Yes	84.9	0.89 (0.56, 1.40)		9.0	1.58 (0.86, 2.90)
HIV disclosure scale to sexual partners	–	1.13 (1.04, 1.22)**	1.10 (1.01, 1.19)*	–	1.04 (0.93, 1.16)
PrEP-related cognitions					
PrEP awareness scale	–	2.09 (1.47, 2.97)***	1.90 (1.32, 2.73)**	–	1.41 (1.01, 1.97)*
PrEP acceptability scale	–	1.17 (1.11, 1.24)***	1.16 (1.09, 1.24)***	–	1.20 (1.07, 1.34)**
Perceived adherence to PrEP scale	–	1.17 (1.10, 1.24)***	1.16 (1.09, 1.23)***	–	1.23 (1.08, 1.41)**
Perceived PrEP benefit in reducing condom use	–	0.94 (0.82, 1.08)	–	–	1.29 (1.07, 1.55)**

†*P* < 0.10, *P < 0.05, **P < 0.01, ***P < 0.001;

*PrEP* Pre-exposure prophylaxis, *STI* sexually transmitted infection, *ORu* univariate odds ratio, *AOR* adjusted odds ratio, odds ratios adjusted by multivariately significant background variables in Table 3, including age, marital status, personal monthly income, and age of first homosexual intercourse;

#### Association between HIV-related characteristics and pay for PrEP

In the univariate analysis, HIV literacy scale (ORu = 1.20; 95% CI: 1.09–1.32) and HIV disclosure scale to sexual partners (ORu = 1.13; 95% CI: 1.04–1.22) were positively associated with participants’ willingness to pay for PrEP. Similar results were found after adjusting for significant background variables. Perception of risk for HIV infection, inconsistent condom use, and multiple sex partnership were not significantly associated with this willingness. None of the above variables was associated with participants’ willingness to pay $283 per month for PrEP. (Table 4).

#### Association between PrEP-related cognitions and pay for PrEP

In the univariate analysis, except for the perceived PrEP benefit in reducing condom use, all other three PrEP-related cognitive variables were positively associated with participants’ willingness to pay for PrEP, including PrEP awareness scale (ORu = 2.09; 95% CI: 1.47–2.97), PrEP acceptability scale (ORu = 1.17; 95% CI: 1.11–1.24), and perceived adherence to PrEP scale (ORu = 1.17; 95% CI: 1.10–0.24). Similar results were found after adjusting for significant background variables.

All four PrEP-related cognitive variables were positively associated with participants’ willingness to pay $283 per month for PrEP, including PrEP awareness scale (ORu/AOR = 1.41; 95% CI: 1.01–1.97), PrEP acceptability scale (ORu/AOR =1.20; 95% CI: 1.07–1.34), perceived adherence to PrEP scale (ORu/AOR =1.23; 95% CI: 1.08–1.41), and perceived PrEP benefit in reducing condom use (ORu/AOR =1.29; 95% CI: 1.07–1.55). (Table 4).

#### Multivariate analysis of factors associated with pay for PrEP

The multivariate stepwise regression model showed that three variables were significantly associated with pay for PrEP, including HIV literacy scale (ORm = 1.12; 95% CI: 1.00–1.25, *p* < 0.05), PrEP awareness scale (ORm = 1.60; 95% CI: 1.10–2.33), and PrEP acceptability scale (ORm = 1.11; 95% CI: 1.00–1.24, p < 0.05). However, the other three variables, overall disclosure of sexual orientation to health professionals, HIV disclosure scale to sexual partners, and perceived adherence to PrEP scale were not selected by the stepwise model.

The multivariate stepwise regression model showed that three variables were significantly associated with participants’ willingness to pay $283 per month for PrEP, including PrEP awareness scale (ORm = 1.35; 95% CI: 0.96–1.90, *p* < 0.1), and PrEP acceptability scale (ORm = 1.19; 95% CI: 1.06–1.33), and perceived PrEP benefit in reducing condom use (ORm = 1.28; 95% CI: 1.07–1.54). However, perceived adherence to PrEP scale was not selected by the stepwise model. (Table 5).

**Table 5:** Summary model of factors associated with pay for PrEP

Items	Pay^a^		Pay $283^b^	
	ORm (95% CI)	*p* value	ORm (95% CI)	*p* value
Health status and service utilization				
Overall disclosure of sexual orientation to health professionals		0.347	N.A.	
HIV-related characteristics				
HIV literacy scale	1.12 (1.00, 1.25)*	0.044	N.A.	
HIV disclosure scale to sexual partners		0.240	N.A.	
PrEP-related cognition				
PrEP awareness scale	1.60 (1.10, 2.33)*	0.015	**1.35 (0.96, 1.90)†**	0.080
PrEP acceptability scale	1.11 (1.00, 1.24)*	0.046	1.19 (1.06, 1.33)**	0.002
Perceived adherence to PrEP scale		0.429		0.247
Perceived PrEP benefit in reducing condom use	N.A		1.28 (1.07, 1.54)**	0.009

†*P* < 0.10, *P < 0.05, **P < 0.01, ***P < 0.001;

*PrEP* Pre-exposure prophylaxis;

*ORm* multivariate odds ratio;

^a^ Four significant background variables (age, marital status, personal monthly income, and age of first homosexual intercourse) were forced entered in the first step, then six variables (overall disclosure of sexual orientation to health professionals, HIV literacy scale, HIV disclosure scale to sexual partners, PrEP awareness scale, PrEP acceptability scale, and perceived adherence to PrEP scale) were put in the multivariate model. The Forward Stepwise (Wald) Method ((Entry: *p* < 0.05, exclude: *p* > 0.10)) was used to select variables in this model;

^b^ Four variabls (including PrEP awareness scale, PrEP acceptability scale, efficacy scale of keeping adherence to PrEP, and perceived benefits of PrEP in reducing condom use) were put in the multivariate model, and the Forward Stepwise (Wald) Method ((Entry: *p* < 0.05, exclude: *p* > 0.10)) was used to select variables in this model;

*N.A* not applicable to this model;

Generally, we found similar results in terms of factors associated with the willing to pay for the generic drug market rate of PrEP (≥85 per month). (Table S-1, S-2, S-3).

## Discussion

The present study found that the majority of the 689 Chinese MSM were unwilling to pay for PrEP: 14.1% would not pay any money and 93.2% would not pay the market rate. We found that PrEP awareness, acceptability, perceived PrEP adherence, and perceived PrEP benefit in reducing condom use were all associated with participants’ willingness to pay for PrEP. Other facilitators of this pay willingness included full disclosure of sexual orientation to health professionals, high HIV literacy, and a high degree of HIV disclosure with sex partners while barriers to this pay willingness included older age and marital status.

The low willingness to pay for PrEP among this sample of MSM in Mainland China was consistent with similar studies conducted in the other regions, including Taiwan, the United States, Cannada, and Thailand [23, 26, 27]. Participants reported a high initial willingness to use PrEP (when it was free) but a low pay willingness, and this discrepancy might be a consequence of PrEP cost and affordability. Consequently, our finding that only 6.8% of participants were willing to pay $283 monthly for PrEP in this study may reflect a more realistic level of PrEP acceptability among Chinese MSM. Policy advocacy and program promotion should take this low pay willingness into consideration. Researchers are suggested to include the willingness to pay in future scales of PrEP acceptability. It is worth noting that, despite the high risk of HIV infection among MSM in general, some MSM may be less risky than others for HIV infection due to various reasons (e.g., consistent condom use) and thus are not in great need of PrEP. Those who are not at high risk of HIV (as an individual) are potentially less likely to want to pay for PrEP, perhaps because the cost outweighs the individual benefit for them.

This study suggests that a strong incentive for the private use of PrEP among MSM is to enable condomless intercourse that is safe from the risk of HIV transmission. Safe sex messages for MSM using PrEP, however, should highlight potential risks of acquiring other STIs when condomless intercourse is practiced.

The present study is subject to several limitations. First, all participants were recruited from a local NGO in Chengdu, and this single recruitment site was likely to have led to a highly selected sample. Findings based on this sample might not be generalizable to all MSM living in Chengdu, MSM living in other parts of China and worldwide. Further studies in other regions of China and worldwide are needed. The cultural perspectives of paying for PrEP should be taken into consideration as well. Second, self-report bias might exist as we asked the participants to recall their STI history and the age of first homosexual intercourse. Social desirability bias might also exist regarding questions on intention to test HIV and sexual behaviors. To reduce such bias, we emphasized to the participants that participation was totally voluntary, and participants were guaranteed that their personal data was highly confidential and inaccessible to third parties. In addition, the questionnaire was self-administrated using an iPad, which would introduce less bias than interviewer-administrated questionnaire using paper-and-pencil. Third, we measured some key variables with a single item, including risk perception of HIV infection and perceived PrEP benefit in reducing condom use. Some self-developed measures (e.g., PrEP awareness scale) present low Cronbach’s alpha coefficients. Further improvements in these measures are suggested, including revising items based on the result of Explanatory Factor Analysis (EFA) and further testing among other samples of MSM living in other places. Fourth, LAI-PrEP is not yet proven efficacious and it is not available anywhere except in efficacy trials currently in the field. It might be problematic by grouping LAI-PrEP with oral daily and oral on-demand PrEP together. Fifth, we did not make detailed differentiation between regular sex partners and casual sex partners when reporting condom use behavior. This might lead to an inaccurate estimation on the association between condom use and pay willingness for PrEP. Last, the present study investigated participants’ willingness (intention) to pay for PrEP, but there might be an intention-behavior gap between this willingness and the actual pay for PrEP (behavior) later, therefore, interpretation of findings should be cautious.

Despite the above limitations, this is one of the first studies to investigate the willingness to pay for PrEP among Chinese MSM. Also, we assessed a comprehensive profile of PrEP-related cognitions with reliable scales, including awareness, acceptability, and perceived adherence to PrEP scale. Some of the investigated factors were novel, including trust with health professionals, HIV communication patterns with sex partners, and an updated scale of HIV literacy. Findings on the low willingness to pay for PrEP indicate that policy support is needed to PrEP implementation and scale-up, such as decrease the cost of PrEP by supporting the development of generic PrEP drugs or cover some cost of PrEP by health insurance plans. Service providers should be trained in identifying high risk subgroups who are more likely to be benefited from PrEP, providing high-quality counseling service to clients who are interested in PrEP, and offering follow-up services to PrEP users. Findings on its associated factors could suggest priority subgroups and inform design of programs tailored in terms of PrEP implementation among MSM in China in the near future. By developing programs that are realistic about attitudes to PrEP, policy makers can ensure that PrEP is properly incorporated into HIV prevention strategies, and ensure that it contributes to realizing the goal of zero new infections in China.

## Conclusion

This study investigated the willingness to pay for PrEP among a less investigated sample of Chinese MSM. We found the overall willingness to pay for PrEP was low, which indicated a great barrier to PrEP implementation and scale-up under the current health care system in China. High PrEP awareness, acceptability, and perceived PrEP adherence were identified facilitators of the pay willingness while none disclosure of sexual orientation to health professionals and low HIV literacy were identified barriers. Programs aiming to promote PrEP pay willingness should pay attention to the important roles of providing enhanced counseling to improve PrEP-related cognitions, delivering accurate HIV/PrEP information to increase health literacy, and decreasing stigma towards sexual minority to develop trust with health professionals. In addition to promote PrEP pay willingness among MSM, efforts should also be made to provide financial assistance to those MSM in need but in low economic affordability to ensure equity. In summary, to promote PrEP uptake in the near future in China, particular efforts should be made to develop supportive policies regarding PrEP drug approval and reimbursement, train staff in providing high-quality PrEP counseling and services, and engage MSM community for PrEP key message delivery.

## Supplementary information

**Additional file 1: Table S-1.** Background characteristics associated with pay $85 for PrEP.

**Additional file 2: Table S-2.** Univariate analysis on factors associated with pay $85 for PrEP.

**Additional file 3: Table S-3.** Summary model of factors associated with pay $85 for PrEP.

## Data Availability

The datasets generated and/or analysed during the current study are not publicly available due to ethical concerns but are available from the corresponding author on reasonable request.

## Abbreviations

PrEP: Pre-exposure Prophylaxis
MSM: Men Who Have Sex with Men
ARV: Antiretroviral
LAI-PrEP: Long-acting Injectable PrEP
STI: Sexually Transmitted Infection
RCT: Randomized Controlled Trial
ORu: Univariate Odds Ratio
ORm: Multivariate Odds Ratio
AOR: Adjusted Odds Ratio
95% CI: 95% Confidence Interval
USD: United States Dollar
