# Supplementary material for: Low willingness to pay for pre-exposure prophylaxis (PrEP) among men who have sex with men (MSM) in China
Source: BMC Public Health. 2020 Mar 16;20:337. doi: 10.1186/s12889-020-08488-w (PMC7077166; doi:10.1186/s12889-020-08488-w)
Supplement: Supplementary file 1 — Additional file 1: Table S-1. Background characteristics associated with pay $85 for PrEP. [file 12889_2020_8488_MOESM1_ESM.docx]

Additional file 1: **Table S-1.** Background characteristics associated with pay $85 for PrEP

| Items | **Pay $85** | |
| --- | --- | --- |
|  | Row% | ORu (95% CI) |
| Age (years old) |  |  |
| <=25 | 28.6 | 1.00 |
| >25 | 25.9 | 0.87 (0.62, 1.22) |
| Highest education obtained |  |  |
| Below than university | 24.1 | 1.00 |
| University or above | 29.4 | 1.31 (0.93, 1.84) |
| Marital status |  |  |
| Single | 29.8 | 1.00 |
| Married | 17.4 | 0.50 (0.28, 0.89)* |
| Having boyfriends | 31.3 | 1.07 (0.73, 1.58) |
| Type of job |  |  |
| Full time | 30.1 | 1.00 |
| Part time | 13.3 | 0.36 (0.12, 1.04)† |
| Unemployed | 23.1 | 0.70 (0.48, 1.01)† |
| Personal monthly income | --- | 1.26 (1.12, 1.42)*** |
| Self-identified sexual orientation |  |  |
| Homosexual | 28.1 | 1.00 |
| Bisexual | 24.4 | 0.83 (0.53, 1.29) |
| Age of first homosexual intercourse (years old) |  |  |
| <21 | 31.2 | 1.00 |
| >=21 | 22.2 | 0.63 (0.45, 0.89)** |

†P<0.10, *P<0.05, **P<0.01, ***P<0.001;

ORu: univariate odds ratio.
