# Supplementary material for: Low willingness to pay for pre-exposure prophylaxis (PrEP) among men who have sex with men (MSM) in China
Source: BMC Public Health. 2020 Mar 16;20:337. doi: 10.1186/s12889-020-08488-w (PMC7077166; doi:10.1186/s12889-020-08488-w)
Supplement: Supplementary file 2 — Additional file 2: Table S-2. Univariate analysis on factors associated with pay $85 for PrEP. [file 12889_2020_8488_MOESM2_ESM.docx]

Additional file 2: **Table S-2.** Univariate analysis on factors associated with pay $85 for PrEP

| Items | **Pay $85** | | |
| --- | --- | --- | --- |
|  | Row% | ORu (95% CI) | AOR (95% CI) |
| **Health status and service utilization** |  |  |  |
| Self-rated health status | --- | 1.06 (0.84, 1.35) | --- |
| The history of STI |  |  |  |
| No | 27.4 | 1.00 | --- |
| Yes | 24.1 | 0.84 (0.44, 1.61) |  |
| HIV testing ever |  |  |  |
| No | 25.4 | 1.00 | --- |
| Yes | 27.5 | 1.12 (0.72, 1.74) |  |
| Intention to test HIV status in the next six months |  |  |  |
| Low intention | 22.9 | 1.00 | --- |
| High intention | 28.9 | 1.37 (0.94, 2.00) |  |
| Overall disclosure of sexual orientation to health professionals | --- | 1.10 (0.91, 1.32) | --- |
| **HIV-related characteristics** |  |  |  |
| Perception of risk for HIV infection | --- | 0.98 (0.84, 1.15) | --- |
| Perception of risk for STI infection | --- | 1.05 (0.90, 1.22) | --- |
| HIV literacy scale | --- | 1.10 (1.00, 1.20)* | 1.05 (0.96, 1.16) |
| Sexual behaviors in the past month |  |  |  |
| Inconsistent condom use |  |  |  |
| No | 25.9 | 1.00 | --- |
| Yes | 30.2 | 1.23 (0.86, 1.78) |  |
| Engage in multiple sex partnership |  |  |  |
| No | 27.0 | 1.00 | --- |
| Yes | 27.4 | 1.01 (0.71, 1.46) |  |
| HIV disclosure scale to sexual partners | --- | 1.11 (1.04, 1.19)** | 1.09 (1.02, 1.17)* |
| **PrEP-related cognitions** |  |  |  |
| PrEP awareness scale | --- | 1.58 (1.29, 1.93)*** | 1.45 (1.17, 1.79)** |
| PrEP acceptability scale | --- | 1.08 (1.02, 1.13)** | 1.08 (1.02, 1.14)** |
| Perceived PrEP adherence scale | --- | 1.12 (1.06, 1.19)*** | 1.12 (1.05, 1.19) ** |
| Perceived PrEP benefit in reducing condom use | --- | 0.94 (0.85, 1.05) | --- |

†P<0.10, *P<0.05, **P<0.01, ***P<0.001;

PrEP: Pre-exposure prophylaxis; STI: sexually transmitted infection;

ORu: univariate odds ratio;

AOR: adjusted odds ratio, odds ratios adjusted by multivariately significant background variables, including marital status, personal monthly income, and age of first homosexual intercourse.
