# Supplementary material for: Low willingness to pay for pre-exposure prophylaxis (PrEP) among men who have sex with men (MSM) in China
Source: BMC Public Health. 2020 Mar 16;20:337. doi: 10.1186/s12889-020-08488-w (PMC7077166; doi:10.1186/s12889-020-08488-w)
Supplement: Supplementary file 3 — Additional file 3: Table S-3. Summary model of factors associated with pay $85 for PrEP. [file 12889_2020_8488_MOESM3_ESM.docx]

Additional file 3: **Table S-3.** Summary model of factors associated with pay $85 for PrEP

| Items | **Pay $85** | |
| --- | --- | --- |
|  | ORm (95% CI) | p value |
| **HIV-related characteristics** |  |  |
| HIV literacy scale |  | 0.743 |
| HIV disclosure scale to sexual partners | 1.08 (1.01, 1.16)* | 0.032 |
|  |  |  |
| **PrEP-related cognition** |  |  |
| PrEP awareness scale | 1.38 (1.11, 1.71)** | 0.004 |
| PrEP acceptability scale |  | 0.938 |
| Perceived PrEP adherence scale | 1.11 (1.01, 1.21)* | 0.031 |

†P<0.10, *P<0.05, **P<0.01, ***P<0.001;

PrEP: Pre-exposure prophylaxis;

ORm: multivariate odds ratio;

Three significant background variables (marital status, personal monthly income, and age of first homosexual intercourse) were forced entered in the first step, then five variables (HIV literacy scale, HIV disclosure scale to sexual partners, PrEP awareness scale, PrEP acceptability scale, and self-efficacy scale of keeping adherence to PrEP) were put in the multivariate model. The Forward Stepwise (Wald) Method ((Entry: p<0.05, exclude: p>0.10)) was used to select variables in this model.
